# Efficacy of adjuvant immunotherapy with cytokine-induced killer cells in patients with locally advanced gastric cancer

**DOI:** 10.1007/s00262-012-1289-2

**Published:** 2012-06-07

**Authors:** Liangrong Shi, Qi Zhou, Jun Wu, Mei Ji, Guojun Li, Jingting Jiang, Changping Wu

**Affiliations:** 1 Department of Tumor Biological Treatment, The Third Affiliated Hospital, Soochow University, 185 Juqian Street, Changzhou, 213003 Jiangsu Province China; 2 Department of Epidemiology, The University of Texas MD Anderson Cancer Center, 1515 Holcombe Blvd, Houston, TX 77030 USA

**Keywords:** Gastric cancer, Cytokine-induced killer cells, Chemotherapy, Adjuvant, Immunotherapy

## Abstract

**Purpose:**

To determine the long-term efficacy of adjuvant immunotherapy with autologous cytokine-induced killer (CIK) cells for locally advanced gastric cancer patients.

**Experimental design:**

One hundred and fifty-one patients with stage III/IV gastric cancer who had undergone gastrectomy were enrolled, assigned to two groups (immunotherapy group versus no immunotherapy group/or control group), and followed.

**Results:**

The 5-year overall survival (OS) and 5-year disease-free survival (DFS) rates for immunotherapy versus control group were 32.4 versus 23.4 % (*P* = 0.071) and 28.3 versus 10.4 % (*P* = 0.044), respectively. For patients with intestinal-type tumors, the 5-year OS and DFS rates were significantly higher for immunotherapy (OS, 46.8 vs. 31.4 % and *P* = 0.045; DFS, 42.4 vs. 15.7 % and *P* = 0.023). In the immunotherapy group, the mean CD3^+^ level, CD4^+^ level, and CD4^+^/CD8^+^ ratio increased from 50.8, 26.5, and 0.9 %, respectively, at baseline to 62.6, 35.0, and 1.4 %, respectively, 1 week after the first CIK-cell treatment, returned to baseline after 2 months, and maintained a higher level (60.7 ± 8.2 %, 34.2 ± 7.1 %, and 1.3 ± 0.3 %, respectively) 2 months after 3 cycles of immunotherapy.

**Conclusions:**

Adjuvant immunotherapy with CIK cells prolongs DFS in patients with locally advanced gastric cancer and significantly improves OS in patients with intestinal-type tumors. Intestinal-type tumors could be selected as an important indication for CIK-cell therapy. This treatment may help improve T-lymphocyte subset distribution and improve the host’s immune functions, but multiple cycles are necessary for long-term therapeutic efficacy.

## Introduction

The global age-adjusted gastric cancer incidence decreased by 15 % since 1985[1]. Nevertheless, it remains an important clinical issue in developed countries. In China, gastric cancer is the leading cause of cancer-related death, and the world-adjusted mortality rate from gastric cancer is the highest in the world [2].

The prognosis of patients with locally advanced gastric cancer is generally dismal; 5-year OS rates are generally 25 % or less even when multimodal treatment strategies involving surgery, perioperative chemotherapy, and radiation are used [3–7]. Most studies to date indicated that adjuvant chemotherapy had little impact on the OS rate. There is currently no standard regimen for postoperative treatment [8]. However, although convincing data are lacking, postoperative chemotherapy based on 5-fluorouracil (5-FU) has been widely used in China.

The immunosuppressed status of patients with cancer has been reported previously [9, 10]. Immunosuppression may be more serious after surgery for the treatment for malignant diseases, including gastric cancer [11]. After adjuvant chemotherapy, the host’s immune functions would be expected to be further damaged because most chemotherapeutic agents are immunosuppressive. There has been a considerable interest in the hypothesis that impaired immunity is common in cancer patients and that tumor may recur unless therapy to reverse immunosuppression is administered together with anticancer treatment [12]. Many reports indicate that adjuvant immunotherapy with immune response cells or biological response modifiers may augment the host immune system, leading to improved survival [13–17]. Several studies published in recent years on gastric cancer suggested a significant improvement in patients receiving immunotherapy with nonspecific immunopotentiators such as polysaccharide K [18], bacille Calmette–Guérin [16], and OK-432 [19, 20]. Adjuvant immunotherapy is expected to be synergistic with surgical resection in the treatment for malignancy [17]. Thus, adjuvant immunotherapy may represent an effective modality to improve the survival rate of patients with gastric cancer.

Treatment with cytokine-induced killer (CIK) cells is one of the promising cellular immunotherapies. It has been demonstrated that CIK cells proliferate abundantly in vitro and can kill tumor cells directly [21]. Furthermore, CIK cells can regulate and increase host cellular immune function in vivo [22]. Therefore, it is biologically plausible to investigate the use of CIK cells for immunotherapy against residual tumor cells. The combination of CIK cells and chemotherapy has been used in clinical practice and has shown potential benefits in patients with recurrent tumors [23].

We previously reported that immunotherapy with CIK cells improved the response rate and increased the 2-year survival rate of patients suffering from advanced gastric cancer [24]. In the study reported here, we compared the long-term effect of adjuvant immunotherapy with CIK cells and chemotherapy only on the survival of patients with locally advanced gastric cancer following gastrectomy, and we investigated the changes in hosts’ cellular immune functions after CIK-cell therapy.

## Materials and methods

### Study patients

All patients who had undergone gastrectomy and were histologically confirmed gastric adenocarcinoma were consecutively recruited through the Department of Tumor Biological Treatment Clinic at The Third Affiliated Hospital, University of Soochow between May 2002 and June 2005, as part of a clinical trial study on gastric adenocarcinoma. These patients were diagnosed and histologically confirmed with stage IIIA, IIIB, or IV (M0) according to the International Union Against Cancer (UICC 2002) TNM system; had to have received 6 cycles of adjuvant chemotherapy based on 5-FU; and had to have had an Eastern Cooperative Oncology Group performance status of 2 or less before adjuvant chemotherapy. The exclusion criteria for eligibility were receipt of adjuvant radiotherapy or other immunotherapy, concurrent active malignancy, and recurrence identified within 6 months after operation. All patients underwent R0 curative gastrectomy with D2 lymph node, that is, N1 and N2, dissection. Indications for total stomach resection were diffuse or mixed-type cancer according to Lauren’s classification and intestinal-type tumor of the middle and upper stomach with a proximal margin of no less than 5 cm. This study was conducted according to the principles of the Declaration of Helsinki and was approved by the Ethics Committee of The Third Affiliated Hospital of Soochow University. All the patients provided informed consent prior to enrollment.

Of the 158 enrolled patients, 151 patients were eligible for the study. If patients who received immmunotherapy with CIK cells were treated as an ‘immunotherapy group’ or ‘treatment group’, the rest without immunotherapy were treated as a ‘no immunotherapy group’ or ‘control group’.

### Induction of CIK cells and determination of their cytotoxic activity

The peripheral blood mononuclear cells (PBMCs) were isolated by Ficoll-Conray density gradient centrifugation, as described previously [25], and were collected using blood cell separators (Baxter, Deerfield, IL, USA). The number of seeded PBMCs is about 1 × 10^7^. After the viability of the PBMCs was assessed by trypan blue exclusion, the PBMCs (2.0 × 10^6^/ml) were plated onto 6-well dishes (Nunc, Roskilde, Denmark) and cultured with Medium I containing RPMI 1640 in the presence of human interferon-gamma (1.0 × 10^6^ U/L, Shanghai Fosun Pharma Co., Shanghai, China); recombinant human interleukin 2 (5.0 × 10^5^ U/L, Shandong Quanguang Pharmaceutical Co., Quanguang, China); 10 % inactivated human serum; 25 mM HEPES; and 2 mM l-glutamine. The cells were incubated in a humidified atmosphere with 5 % CO_2_ at 37 °C. After 24 h, monoclonal antibody against CD3 (100 μg/L, Antibody Diagnostic Inc., New York, NY, USA) and interleukin-1 alpha (1.0 × 10^5^ U/L, Promega Biological Products, Ltd., Shanghai, China) were added. After another 48 h, the supernatant was removed by aspiration and the cells were cultured in Medium II (Medium I without interferon-gamma). The medium was replaced every 3 days. On days 1, 7, 14, 21, and 28, the cells were identified and sorted by flow cytometry (Beckman-Coulter, Fullerton, CA, USA). Before infusion, the viability of CIK was tested by the dye-exclusion with no less than 95 % viable cells. The cytotoxic activity of the CIK cells was determined by co-incubation with the natural killer cell-sensitive K562 cell line (American Type Culture Collection, Manassas, VA, USA) as described previously [24].

### Treatments

Before enrollment in this study, all patients received 6 cycles of multidrug adjuvant chemotherapy based on 5-FU. The patients in the immunotherapy group received at least 3 cycles of CIK-cell therapy after adjuvant chemotherapy unless recurrence was ascertained. The patients in the control group did not receive immunotherapy. The first cycle was started 6 weeks after the end of adjuvant chemotherapy, and the subsequent cycles were started at the intervals of 8–12 weeks. More than 1 × 10^9^ CIK cells were transfused into patients within 1 h every second day via superficial vein. Five transfusions were defined as 1 cycle. When the patients in either the immunotherapy group or the control group were diagnosed with recurrence, second-line chemotherapy or palliative surgery was performed.

### Follow-up

The postoperative baseline and follow-up investigations were documented. The baseline assessments included a complete medical history, physical examination, and diagnostic imaging, including abdominal ultrasonography or computed tomography and chest radiography.

Follow-up was the same for the immunotherapy and control groups, and performed every 3 months for the first 2 years after CIK-cell therapy, every 6 months for the next 3 years, and yearly thereafter. Follow-up consisted of physical examination, blood counting, liver function, CEA level, abdominal ultrasonography or computed tomography, and chest radiography. Gastroscopy was also performed for patients in whom regional recurrence was suspected. In patients with recurrence and patients who died, the site and date of the first recurrence and the date of death were recorded. Disease recurrence was diagnosed by physical and radiological examinations, and routine histological examinations were carried our as needed. Patients were followed up until they were lost to follow-up or died or until October 25, 2010.

In the immunotherapy group, lymphocyte subsets were detected by flow cytometry in peripheral blood 2 weeks after the completion of adjuvant chemotherapy (baseline) and 1 and 8 weeks after the start of every cycle of CIK-cell therapy. In the control group, lymphocyte subsets were detected by flow cytometry 6 months after the completion of adjuvant chemotherapy.

### Statistical analysis

Differences in distribution of selected demographic and clinical characteristics between the immunotherapy and control groups were evaluated using the Student’s *t* test and χ^2^ test. Lymphocyte subsets before and after CIK therapy were compared by the paired-sample *t* test, and continuous data at multiple time points in the same individual were analyzed by repeated-measures analysis of variance. The main end point was overall survival (OS). Secondary end points were disease-free survival (DFS) and cellular immune response. OS and DFS were defined as the time from the date of operation to the date of death from any cause or the first occurrence of a neoplastic event (relapse or second malignancy) or the date of the last follow-up. Participants who were alive or recurrence free at the end of the study period or lost to follow-up were censored. OS and DFS curves were estimated using the Kaplan–Meier method, and the Cox’s model for hazard ratio (HR) and 95 % confidence interval (CI) was performed for comparison of the immunotherapy with control groups. Multivariable model was adjusted with possible underlying variables that were statistically significant in the univariate analysis. *P* < 0.05 was considered statistically significant. Data were analyzed using SPSS software (version 13.0, SPSS Inc., Chicago, IL, USA.).

## Results

### Patient characteristics

A total of 158 patients were recruited for the study at The Third Affiliated Hospital of Soochow University from April 2002 to June 2005. Seven patients were excluded as 4 patients in immunotherapy group were refused further CIK-cell therapy after the first cycle; the other 3 were in the control group and received other immunotherapies after enrollment. Therefore, the final analyses included 151 eligible patients, 77 in the control group, and 74 in the immunotherapy group. There were no significant differences in demographic and clinical characteristics between the 2 groups except that the proportion of female patients was higher in the immunotherapy group (*P* = 0.037) (Table 1).

**Table 1:** Demographic and clinical characteristics of patients

Characteristic	Total (*n* = 151)	Chemotherapy only (control) (*n* = 77)	Immunotherapy (*n* = 74)	*P*
Sex, male/female	101/50	58/19	43/31	0.037
Age (year), median ± SE	57.0 ± 1.2	56 ± 1.5	58 ± 2.1	0.692
ECOG performance status				
0/1	136	69	67	0.915
2	15	8	7	
Surgical procedure				
Partial gastrectomy	82	39	40	0.745
Total gastrectomy	69	38	34	
Location of tumor				
Upper	39	18	21	0.772
Middle	41	22	19	
Lower	71	37	34	
Pathological type of tumor				
Intestinal type	98	51	47	0.598
Diffuse	42	22	20	
Mixed type	11	4	7	
Histologic differentiation				
Well differentiated	41	23	18	0.644
Poorly differentiated	78	37	41	
Signet-ring cell	32	17	15	
UICC stage				
IIIA	46	24	22	0.837
IIIB	72	35	37	
VI	33	18	15	
Chemotherapy regimen				
Cisplatin	28	18	10	0.101
Oxaliplatin	79	34	45	
Docetaxel	44	25	19	

SE indicates standard error; *ECOG* Eastern Cooperative Oncology Group, *UICC* International Union Against Cancer

### Induction of CIK cells

The proliferation and phenotypes of the PBMCs after CIK induction varied between individuals. The cell number increased more than 100-fold on average after 14-day incubation. The number of CIK (CD3^+^CD56^+^) cells increased greatly, from 400-fold to more than 1,400-fold depending on the individual, with an average of 700-fold. The number of CIK cells peaked at day 14 and then slightly decreased by day 28. All CIK cells administered met the following criteria: The percentages of CD3^+^ and CD8^+^ cells exceeded 70 and 40 %, respectively, and CD3^+^CD56^+^ cells were no less than 30 %. The cytotoxic activity of the CIK cells was highest at day 14 (70.5 ± 5.2 %). These results are similar to those in our previously published articles [24, 26]. The final cell products were assessed for viability by the dye-exclusion test and checked twice for possible contamination by bacteria, fungi, and endotoxins. At least 1 × 10^9^ CIK cells were harvested and transfused into patients within 1 h every second day since day 14 to day 22 for 5 times.

### OS and DFS

Three patients (1 in the immunotherapy group and 2 in the control group) were not followed up, and they were censored at the time of their last visit. Four patients in the immunotherapy group received only 2 cycles of CIK-cell therapy because the recurrence was diagnosed within 6 months after adjuvant chemotherapy. These patients were not excluded from the study in order to keep balance between the 2 groups.

The median follow-up period was 50.5 months (range, 18–82 months). By the end of follow-up, 137 patients (90.7 %) had died, and 143 patients (94.7 %) had been diagnosed with recurrence. The causes of death and sites of recurrence are shown in Table 2. Of the patients with recurrence, 54.5 % had a hematogenous recurrence which was most frequent, 27.3 % had a peritoneal recurrence, 19.6 % had a lymphatic recurrence, 18.2 % had a locoregional recurrence, and 19.6 % had multiple forms of recurrence. The hematogenous recurrence rate of the immunotherapy group was 50.0 %, which was moderately lower than that of the control group (58.7 %); however, the difference between the groups was not significant. Tumor-related deaths accounted for 78.5 % of the deaths in the immunotherapy group and 88.9 % of the deaths in the control group. Univariate analysis showed that the following factors were associated with improved OS: lower grade (*P* = 0.000), smaller tumor size (*P* = 0.007), younger age (*P* = 0.000), lower tumor location (*P* = 0.007), earlier tumor UICC stage (*P* = 0.000), and partial gastrectomy (*P* = 0.001). In contrast, sex, chemotherapy regimen, and performance status did not significantly influence OS.

**Table 2:** Death (with causes) and recurrence (with sites) of patients

Characteristic	Total (*n* = 151)	Chemotherapy only (control) (*n* = 77)	Immunotherapy (*n* = 74)
Death	137 (90.7)	72 (93.5)	65 (87.8)
Tumor related	115 (83.9)	64 (88.9)	51 (78.5)
Intercurrent disease	8 (5.8)	3 (4.2)	5 (7.7)
Second malignancy	5 (3.6)	2 (2.8)	3 (4.6)
Unknown cause	9 (6.6)	3 (4.2)	6 (9.2)
Site of recurrence^a^			
Hematogenous	78 (54.5)	44 (58.7)	34 (50.0)
Peritoneal	39 (27.3)	19 (25.3)	20 (29.4)
Lymphatic	28 (19.6)	14 (18.7)	14 (20.6)
Locoregional	26 (18.2)	12 (16.0)	13 (19.1)

^a^Multiple recurrences are included

**Fig. 1:**
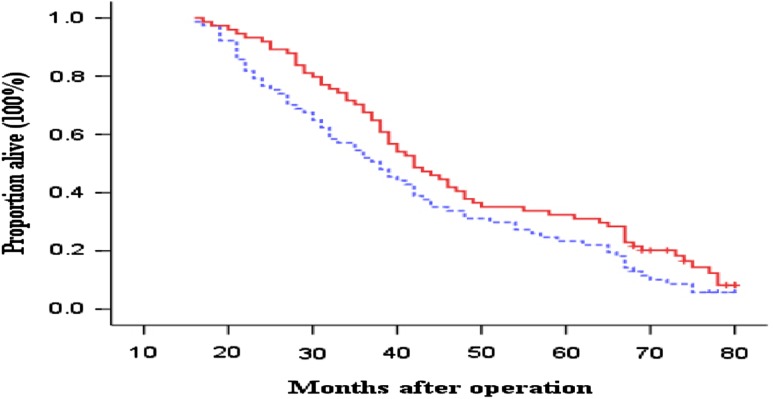
Kaplan–Meier estimates for overall survival (OS) for patients. *Continuous line* immunotherapy group; *dotted line* chemotherapy-only group (control). Log-rank: *P* = 0 0.071

**Fig. 2:**
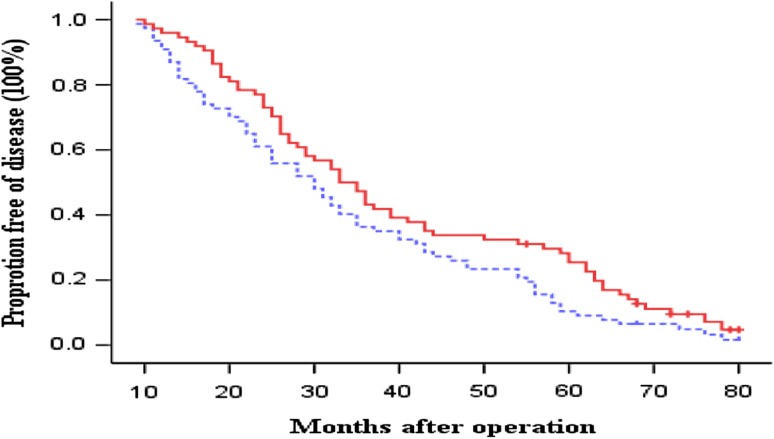
Kaplan–Meier estimates for disease-free survival (DFS) for patients. *Continuous line* immunotherapy group; *dotted line* chemotherapy-only group (control). Log-rank: *P* = 0.044

OS and DFS curves between the immunotherapy and control groups are presented in Figs. 1 and 2, respectively. The patients in immunotherapy group had a borderline significantly and significantly better OS and DFS than the patients in control group (log-rank, *P* = 0.071 for OS and *P* = 0.044 for DFS), respectively. The 3- and 5-year OS rates were 54.5 and 23.4 % in the control group versus 67.6 and 32.4 % in the immunotherapy group. The median OS durations were 42.1 months in the control group and 48.1 months in the immunotherapy group. In contrast, the 3- and 5-year DFS rates were 36.4 and 10.4 % in the control group versus 47.3 and 28.3 % in the immunotherapy group. The median DFS durations were 34.1 months in the control group and 40.4 months in the immunotherapy group. Furthermore, compared with the patients in the control group, the patients in immunotherapy group had borderline and significantly reduced risk of overall death and recurrence (HR, 0.78; 95 % CI, 0.53–1.04 for overall death and HR, 0.72; 95 % CI, 0.52–0.99 for recurrence), respectively.

**Fig. 3:**
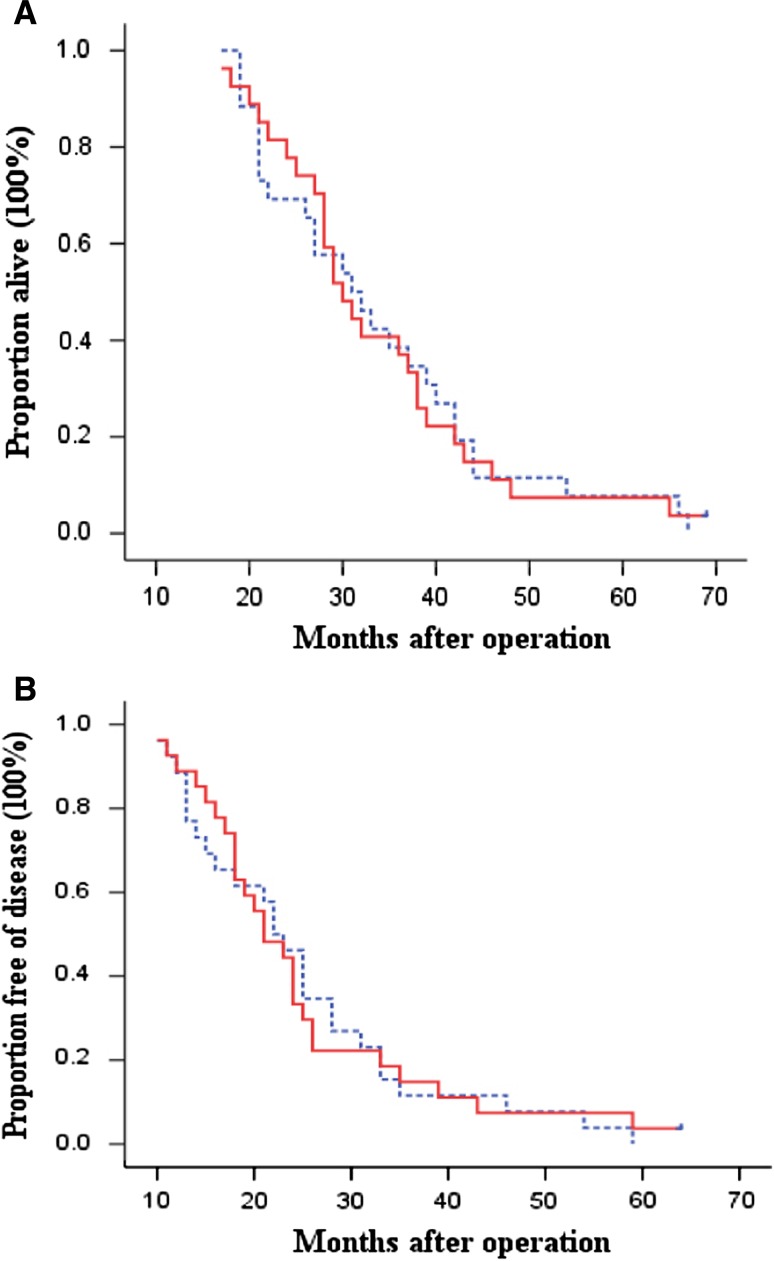
Kaplan–Meier estimates for OS (**a**) and DFS (**b**) for patients with diffuse or mixed-type tumors. *Continuous line* immunotherapy group; *dotted line* chemotherapy-only group (control). Log-rank: *P* = 0.970 for OS and *P* = 0.962 for DFS

**Fig. 4:**
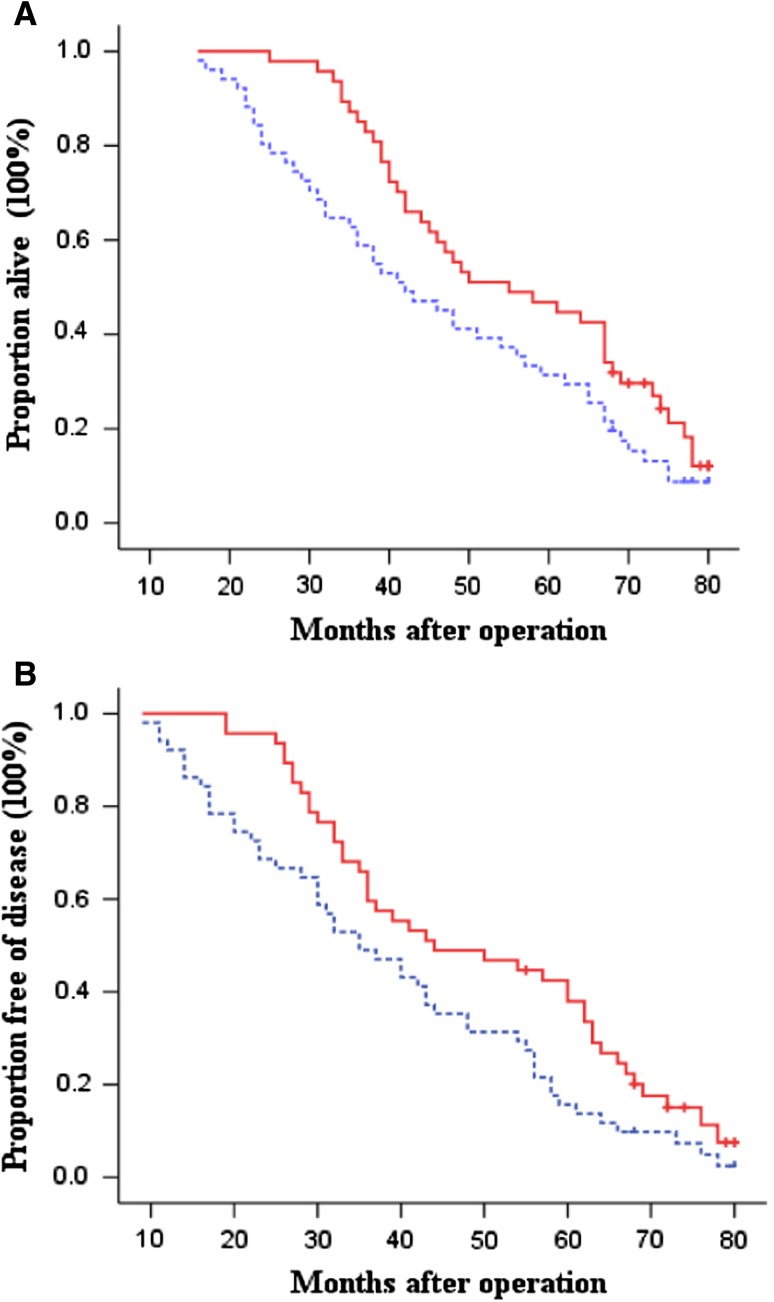
Kaplan–Meier estimates for OS (**a**) and DFS (**b**) for patients with intestinal-type tumors. *Continuous line* immunotherapy group; *dotted line* chemotherapy-only group (control). Log-rank: *P* = 0.045 for OS and *P* = 0.023 for DFS

A retrospective subgroup analysis of patients by Lauren’s histological type showed that patients with diffuse or mixed-type tumors seemed not to benefit from adjuvant immunotherapy. The OS and DFS were not significantly different between the 2 groups (log-rank, *P* = 0.970 for OS and *P* = 0.962 for DFS, Fig. 3). Within this subgroup, the 3- and 5-year OS rates were 38.5 and 7.7 % in the control group versus 40.7 and 7.4 % in the immunotherapy group. The 3- and 5-year DFS rates were 11.5 and 0.0 % in the control group and 14.8 and 3.7 % in the immunotherapy group. In addition, there was no significant difference in risk of overall death and recurrence between the control group and the immunotherapy group (HR, 1.01; 95 % CI, 0.57–1.74 for overall death and HR, 0.98; 95 % CI, 0.57–1.69 for recurrence). However, for the patients with intestinal-type tumors, not only OS but also DFS was significantly better in the immunotherapy group than in the control group (log-rank, *P* = 0.045 for OS and *P* = 0.023 for DFS, respectively, Fig. 4). The 3- and 5-year OS rates were 62.7 and 31.4 % in the control group versus 87.2 and 46.8 % in the immunotherapy group. The 3- and 5-year DFS rates were 49.0 and 15.7 % in the control group versus 66.0 and 42.4 % in the immunotherapy group. The patients in immunotherapy group had significantly reduced risk in overall death and recurrence compared with the patients in the control group (HR, 0.65; 95 % CI, 0.43–1.00 for overall death and HR, 0.62; 95 % CI, 0.41–0.95 for recurrence).

### Cellular immune response after CIK-cell therapy

We evaluated the cellular immune response in the immunotherapy group by analyzing changes in lymphocyte subsets between baseline (2 weeks after adjuvant chemotherapy) and various points during immunotherapy shown in Table 3. At baseline, the percentages of CD3^+^, CD4^+^ cells, and the CD4^+^/CD8^+^ ratio were 50.8 ± 8.5 %, 26.5 ± 6.1 %, and 0.9 ± 0.4, respectively. One week after the end of the first CIK-cell treatment cycle, the percentages of CD3^+^, CD4^+^ cells, and the CD4^+^/CD8^+^ ratio were significantly higher (62.6 ± 11.3 %, 36.0 ± 6.6 %, and 1.4 ± 0.3, respectively). By 2 months after the end of the first treatment cycle, the values declined to near baseline values (51.8 ± 9.2 %, 28.0 ± 7.6 %, and 1.0 ± 0.2 %, respectively). Two months after the end of the third cycle of CIK-cell treatment, values increased to 60.7 ± 8.2 %, 34.2 ± 7.1 %, and 1.3 ± 0.3, respectively, which was significant higher than the baseline. Additionally, the changes of immunologic data in the different subgroups were compared according to Lauren’s histological type. Compared to the patients with intestinal-type cancer, similar cellular immune responses were observed in the patients with diffuse-type and mixed-type cancer.

**Table 3:** Comparison of cellular immune responses before and after CIK therapy

Lymphocyte phenotype	Before CIK therapy	1 week after 1st CCT^a^	2 months after 1st CCT	2 months after 3rd CCT
CD3^+^	50.8 ± 8.5	62.6 ± 11.3*	51.8 ± 9.2	60.7 ± 8.2*
CD4^+^	26.5 ± 6.1	36.0 ± 6.6	28.0 ± 7.6	34.2 ± 7.1
CD4^+^/CD8^+^	0.9 ± 0.4	1.4 ± 0.3*	1.0 ± 0.2	1.3 ± 0.3*

*CIK* cytokine-induced killer

*P < 0.05 compared to before CIK therapy

^a^ *CCT* Cycle of CIK therapy

### Side effects of CIK-cell transfusion

A total of 351 cycles of CIK-cell therapy were performed. Two patients received 2 cycles, 18 patients received 3 cycles, 32 patients received 4–5 cycles, 22 patients received more than 6 cycles, and the rest of 77 patients were in the control group. During and after each of the 351 cycles of CIK-cell therapy that were administered, side effects, including fever, chills, headache, rash, nausea and vomiting, diarrhea, shock, abnormalities of routine blood test, hepatic dysfunction, and renal dysfunction, were recorded. The most common side effects were fever (20.8 %), chills (15 %), headache (10 %), rash (5 %), and nausea and vomiting (5 %). There were no instances of diarrhea, shock, abnormalities of routine blood test, hepatic dysfunction, or renal dysfunction (Table 4). All of the side effects were resolved without intervention within 24 h or were treated successfully by simple allopathy, such as anti-inflammatory treatment, anti-allergy treatment, and antiemetic treatment.

**Table 4:** Side effects of CIK-cell therapy (351 cycles)

Side effects	Fever	Chills	Headache	Rash	Nausea and vomiting	Shock
Yes	73 (20.8)	52 (14.8)	35 (10.0)	18 (5.10)	18 (5.10)	0 (0.00)
No	278 (79.2)	299 (85.2)	316 (90.0)	333 (94.9)	333 (94.9)	100 (100)

*CIK* cytokine-induced killer

Values in table are numbers and percentages of cycles in which side effects were observed

## Discussion

In this study, we assessed the efficacy of adoptive immunotherapy with CIK cells in patients with locally advanced gastric cancer after gastrectomy. To our knowledge, no studies of adjuvant adoptive immunotherapy with immune response cells for gastric cancer have previously been reported. Studies have shown that immune response cells such as lymphokine-activated killer cells [27], tumor-infiltrating lymphocytes [28], anti-CD3 monoclonal antibody-induced killer cells [29], and CIK cells may kill tumor cells directly with high proliferative activity [30]. These cells are non-major histocompatibility complex-restricted in target cell recognition and killing [31]. Interestingly, CIK cells have also been shown to be effective against multidrug-resistant and FasL-positive malignant cells [22, 32]. Moreover, CIK cells can regulate and increase host cellular immune function in vivo by secretion of cytokines, such as interferon-gamma, and a number of chemokines, including RANTES, MIP-1α, and MIP-1β [22, 33]. Because of their inherently high antitumor activity, CIK cells represent one of the promising cellular immunotherapies. Studies have shown that CIK cells from tumor patients (autologous CIK cells) have a high proliferation rate and cytotoxic activity in vitro and have shown the efficacy and safety of transfusing such cells to patients with advanced cancer [24, 26, 34, 35].

In a previous study [24], we have reported the number of CIK-cell therapy to cancer-related death in gastric cancer, showing significant differences in the survival for 156 gastric cancer patients. In the current study, we evaluated the effect of adjuvant immunotherapy with CIK cells after chemotherapy on survival of patients with locally advanced gastric cancer. In order to better balance between the two groups, we set the baseline at the end of six cycles of adjuvant chemotherapy. Therefore, both the patients with early stage (stage I/II) and those without completion of six cycles of adjuvant chemotherapy or recurrence within 6 months after surgery were excluded. We found an improvement of 9 % in 5-year OS rate (*P* = 0.071) and an improvement of 17.9 % in 5-year DFS rate (*P* = 0.044) in the patients receiving adjuvant immunotherapy. Moreover, the stratified analysis by Lauren’s histological type revealed that patients with intestinal-type tumors, but not those with diffuse or mixed-type tumors, responded to immunotherapy. For the patients with intestinal-type tumors, not only OS but also DFS was significantly longer in the immunotherapy group than in the control group. The interpretation of these findings should be treated with caution as it is an exploratory analysis within a subgroup.

Interestingly, response of patients with intestinal-type tumors to immunotherapy was observed in another study [16]. In that study, a nonspecific immunopotentiator, bacille Calmette–Guérin (BCG), was added to chemotherapy. Treatment with BCG was started within 2 weeks after the beginning of chemotherapy and continued for 2 years or until death. The study indicated that adjuvant immunotherapy (BCG + FAM) might prolong the survival of gastric cancer patients after gastrectomy, in particular, in patients with pT2/T3 tumors and intestinal-type primary tumors [16]. In agreement with what has been suggested by previous data [36, 37], our study indicated that Lauren’s histological type is an important prognostic factor, while diffuse and mixed-type tumors are associated with an invasive growth pattern and short survival time [38]. It is well known that neither nonspecific immunopotentiators nor immune response cells can improve the host’s immune system function immediately after a short treatment procedure. This may be an important reason why patients with diffuse and mixed-type tumors do not benefit from immunotherapy—disease advances rapidly or patients die before immunotherapy can have an important effect.

In patients with advanced stages of cancer, the normal immune response is frequently observed to be altered or impaired [39]. Typically, immune function is evaluated by using parameters reflecting either functional or numerical changes of T lymphocytes. The measurement of T-lymphocyte subsets has been reported to be a useful clinical indicator of immunosuppression in a number of disease states [40]. In this study, we found that the percentage of CD3^+^ cells and the ratio of CD4^+^ to CD8^+^ cells were lower than the normal reference values. Similar results have been reported previously by others [11]. We also found that the percentages of CD3^+^, CD4^+^ cells, and the ratio of CD4^+^/CD8^+^ were significantly higher 1 week after the first cycle of CIK-cell therapy but had declined markedly at 2 months. However, 2 months after 3 cycles of immunotherapy, these values maintained a higher level than the baselines. These results indicate that CIK-cell therapy is helpful to improve the T-lymphocyte subset distribution, but the improvement resulting from a single cycle of therapy is short-lived. Therefore, to gain long-term therapeutic efficacy, multiple cycles of therapy should be administered.

At present, convincing data from large-scale clinical studies of adjuvant chemotherapy for gastric cancer are lacking. There is no standard regimen for adjuvant chemotherapy, although chemotherapy has been found to be active in the treatment for unresectable or metastatic gastric cancer in China. As the main purpose of this study was to evaluate the efficacy of sustained CIK-cell therapy after adjuvant chemotherapy, we set up a baseline at the end of 6 cycles of adjuvant chemotherapy. This could be a limitation of this study without a standard regimen of adjuvant chemotherapy. In addition, a selection bias was another limitation of this study because this was a hospital-based study, and a limited number of patients in each group may not represent the patient population from which the patients arose. Therefore, larger and well-designed studies are needed to confirm our findings. As described previously by others [24, 26, 36], we also observed that CIK-cell therapy was well tolerated. CIK-cell transfusion was associated with no severe side effects, and the slight side effects that were noted, such as chills, fever, headache, and nausea and vomiting, were all eliminated by moderate treatment.

In conclusion, these results indicate that CIK-cell therapy is helpful to improve the host’s immune function after chemotherapy, but multiple cycles are necessary for long-term therapeutic efficacy. Adjuvant immunotherapy with CIK cells can prolong DFS in patients with locally advanced gastric cancer who have undergone gastrectomy and may have a noticeable impact on OS in patients with intestinal-type tumors in particular.
